# Opportunities and Challenges of Predictive Approaches for the Non-coding RNA in Plants

**DOI:** 10.3389/fpls.2022.890663

**Published:** 2022-04-14

**Authors:** Dong Xu, Wenya Yuan, Chunjie Fan, Bobin Liu, Meng-Zhu Lu, Jin Zhang

**Affiliations:** ^1^State Key Laboratory of Subtropical Silviculture, College of Forestry and Biotechnology, Zhejiang A&F University, Hangzhou, China; ^2^Shenzhen Branch, Guangdong Laboratory of Lingnan Modern Agriculture, Genome Analysis Laboratory of the Ministry of Agriculture and Rural Affairs, Agricultural Genomics Institute at Shenzhen, Chinese Academy of Agricultural Sciences, Shenzhen, China; ^3^State Key Laboratory of Tree Genetics and Breeding, Research Institute of Tropical Forestry, Chinese Academy of Forestry, Guangzhou, China; ^4^Jiangsu Key Laboratory for Bioresources of Saline Soils, Jiangsu Synthetic Innovation Center for Coastal Bio-agriculture, School of Wetlands, Yancheng Teachers University, Yancheng, China

**Keywords:** non-coding RNA, bioinformatics tools, experimental technology, deep learning, miRNA

## Introduction

Non-coding RNA (ncRNA) is a key regulatory RNA with limited abilities of coding potential (Liu et al., 2015). To date, a variety of ncRNAs have been identified in plants, which can be classified into three groups according to their sequence length: small RNAs <50 nucleotides (nt), such as microRNAs (miRNAs); long non-coding RNAs (lncRNAs) with arbitrarily longer than 200 nt; and intermediate-size ncRNA between small RNAs and lncRNA in length (Wang et al., 2014). In addition, the process of back splicing also produces a class of covalently closed RNA molecules called exonic circular RNAs (circRNAs).

Different types of ncRNAs show diverse mechanisms of action. miRNAs typically degrade their target genes by binding to their transcripts at the post-transcriptional level (Chipman and Pasquinelli, 2019). However, lncRNAs function at transcriptional, post-transcriptional and epigenetic levels by interacting with macromolecules (Wu et al., 2020). Whiles circRNAs can act as miRNA/protein sponges, or regulate alternative splicing or transcription (Lai et al., 2018). These ncRNAs with different mechanisms of action form a complex regulatory network to jointly regulate plant growth, development and stress response. For instance, *Vvi-miPEP171d1* regulates adventitious root formation in grapevine (Chen et al., 2020). And miRNA frameworks have been proved to be important for the flower induction in apple (Fan et al., 2018). In addition to regulation of self-life activities, ncRNAs also play a role in plant-to-plant communication. Exogenous *miR399* and *miR156* can trigger RNA interference to repress the expression of PHOSPHATE OVERACCUMULATOR 2 (*PHO2*) and SQUAMOSA-PROMOTER BINDING PROTEIN-LIKE 9 (*SPL9*) in plants (Betti et al., 2021). Moreover, ncRNAs can even act as a bridge between plants and other species. For example, two plant viruses, *barley yellow dwarf virus* and *red clover necrotic mosaic virus*, can express small subgenomic (sg) RNAs to attenuate host translation by binding translation initiation factor eIF4G (Miller et al., 2016). Expressing double-strand (ds) RNA in maize can effectively reduce feeding damage of western corn rootworm by triggering its RNA interference (Baum et al., 2007). In summary, ncRNAs play important roles in plant activities, and their regulatory functions are the basis for plant growth, development and survival.

The regulatory roles of ncRNAs make them important tools for adjusting gene expression and studying gene functions. For example, the artificial miRNA *pAmiRNA156h-PDSh* can effectively decline the expression of phytoene desaturase (*MdPDS*) in apple (Charrier et al., 2019). Overexpression of *amiRNA-319a-HaEcR* in tomato can effectively silence the *ECDYSONE RECEPTOR* (*HaEcR*) gene to reduce the survival of *Helicoverpa armigera* (Yogindran and Rajam, 2021). From this point of view, exploring new ncRNAs and elucidating the regulatory mechanism of ncRNAs will have profound impacts on plant research. Here, our emphasis is put on recognition technologies of ncRNAs in plants, including bioinformatic tools and experimental technologies.

## Bioinformatics Tools

With the release of more and more genomic information of diverse plants, it provides a key basis for the discovery of novel ncRNAs. In addition, knowledge about the function, structure and conservation of ncRNAs is accumulating, which can help us distinguish different types of ncRNAs. Since ncRNAs were reported, at least 39 bioinformatics websites and softwares have been established, of which 40.5% (13 websites and 4 softwares) were released in the past 5 years (Table 1). Furthermore, 41 deep learning models were developed in the past 3 years (Table 1). These tools can be divided into three classes.

**Table 1:** Bioinformatics tools in ncRNAs analysis.

**(1) Websites and softwares^a^**		
**Tools**	**Websites**	**Main function**
**(1.1) Database**		
PLncDB	http://www.tobaccodb.org/plncdb/	Long non-coding RNA Database
Rfam	http://rfam.xfam.org/search	RNA sequence-family database
PlantcircBase	http://ibi.zju.edu.cn/plantcircbase/index.php	Predict circRNAs
RNAcentral	https://rnacentral.org/	ncRNA database
PmiRExAt	http://pmirexat.nabi.res.in/	miRNA-expression database
NONCODE	http://www.noncode.org/index.php	ncRNA database
PNRD	http://structuralbiology.cau.edu.cn/PNRD/index.php	miRNA database
scaRNAbase	http://gene.fudan.edu.cn/snoRNAbase.nsf	sno/scaRNA database
**(1.2) Prediction tools**		
miRDeep-P2	https://sourceforge.net/projects/mirdp2/	Analyze miRNA transcriptome
miRBase	https://www.mirbase.org/search.shtml	Contain miRNAs and precursors
psRNATarget	https://www.zhaolab.org/psRNATarget/analysis	Predict target genes of small RNA
TarBase	http://carolina.imis.athena-innovation.gr/diana_tools/web/index.php?r=tarbasev8%2Findex	miRNA-target interactions
PeTMbase	http://tools.ibg.deu.edu.tr/petmbase/	miRNA-target mimics
RNAComposer	http://rnacomposer.ibch.poznan.pl/	Predict 3D structure of ncRNA
COME	https://github.com/lulab/COME	Annotate lncRNAs
comPARE	https://mpss.danforthcenter.org/tools/mirna_apps/comPARE.php	Predict miRNAs and their targets
comTAR	http://rnabiology.ibr-conicet.gov.ar/comtar/	Evolutionary analysis of miRNA and target
plantDARIO	http://plantdario.bioinf.uni-leipzig.de/index.py	Predict ncRNA from RNA-seq data
miRNEST	http://rhesus.amu.edu.pl/mirnest/copy/	miRNAs and targets
PhasiRNAnalyzer	https://cbi.njau.edu.cn/PPSA/	Identify phasiRNAs and their target genes
miRTarBase	https://mirtarbase.cuhk.edu.cn/miRTarBase/miRTarBase_2019/php/index.php	Interaction between miRNAs and their target genes
NPInter	http://bigdata.ibp.ac.cn/npinter4/	Interaction between ncRNAs and biomolecules
RNAshapes	https://bibiserv.cebitec.uni-bielefeld.de/rnashapes	Predict ncRNA structure
RNAcon	http://crdd.osdd.net/raghava/rnacon/	Predict and classify the ncRNAs
miR-PREFeR	https://github.com/hangelwen/miR-PREFeR	Predict miRNAs and precursors
CNCI	https://github.com/www-bioinfo-org/CNCI	Classify lncRNAs
CPAT	http://rna-cpat.sourceforge.net/	Annotate lncRNAs
Infernal	http://eddylab.org/infernal/	Predict ncRNA-secondary sequences
PsRobot	http://omicslab.genetics.ac.cn/psRobot/index.php	Predict stem-loop structure and target of ncRNA
NUPACK	http://www.nupack.org/partition/new	Analyze and design ncRNA structures
MiSolRNAdb	http://www.misolrna.org/	Map position of miRNA and targets
TAPIR	http://bioinformatics.psb.ugent.be/webtools/tapir/	Predict binding sites of miRNA and target
RNAz	https://www.tbi.univie.ac.at/software/RNAz/	Predict ncRNA secondary structures
CentroidAlign	http://www.ncrna.org/software/centroidalign/	Multiple alignments of ncRNAs
CleaveLand4	https://github.com/MikeAxtell/CleaveLand4/ blob/master/CleaveLand4.pl	Predict the binding sites of miRNAs in target
RNAfold	http://rna.tbi.univie.ac.at//cgi-bin/RNAWebSuite/RNAfold.cgi	Provide information of ncRNA secondary structures
fRNAdb	https://dbarchive.biosciencedbc.jp/en/frnadb/download.html	NcRNA sequences, prediction tools
Randfold	https://github.com/erbon7/randfold	Predict secondary structures of ncRNAs
Mfold	http://www.unafold.org/mfold/applications/rna-folding-form.php	Predict the nucleic acid folding and hybridization
**(2) Deep learning models**		
**Models**	**Main function**	**References**
**(2.1) Finding novel ncRNAs or classification**		
ncRDense	ncRNA classification	Chantsalnyam et al., 2021
linc2function	lncRNA identification	Ramakrishnaiah et al., 2021
ncDLRES	ncRNA identification	Wang et al., 2021b
ncRDeep	ncRNA classification	Chantsalnyam et al., 2020
2L-piRNADNN	piRNA identification	Khan et al., 2020
ncPro-ML	ncRNA promoter identification	Tang et al., 2020
circDeep	circular RNA classification	Chaabane et al., 2020
PredLnc-GFStack	ncRNA identification	Liu et al., 2019
LncADeep	lncRNA identification	Yang et al., 2018
nRC	ncRNA classification	Fiannaca et al., 2017
DARIO	ncRNA identification	Fasold et al., 2011
**(2.2) ncRNA-biomolecular interaction**		
NPI-RGCNAE	ncRNA–protein interaction	Yu et al., 2021
DeepLPI	ncRNA–protein interaction	Shaw et al., 2021
PRPI-SC	lncRNA–protein interaction	Zhou et al., 2021
LGFC-CNN	lncRNA–protein interaction	Huang et al., 2021
Capsule-LPI	lncRNA–protein interaction	Li et al., 2021
EDLMFC	ncRNA–protein interaction	Wang et al., 2021a
NPI-GNN	ncRNA–protein interaction	Shen Z. A. et al., 2021
PmliPEMG	miRNA–lncRNA interaction	Kang et al., 2021
lncIBTP	lncRNA-biomolecule interaction	Zhang et al., 2021
RPI-SE	ncRNA–protein interaction	Yi et al., 2020
DRPLPI	lncRNA–protein interaction	Wekesa et al., 2020c
HFC-RPI	ncRNA–protein interaction	Dai et al., 2020
GPLPI	ncRNA–protein interaction	Wekesa et al., 2020b
LPI-DL	lncRNA–protein interaction	Wekesa et al., 2020a
LPI-CNNCP	lncRNA–protein interaction	Zhang S. W. et al., 2020
CIRNN	miRNA–lncRNA interaction	Zhang P. et al., 2020
LncMirNet	miRNA–lncRNA interaction	Yang et al., 2020
PmliPred	miRNA–lncRNA interaction	Kang et al., 2020
LMI-DForest	miRNA–lncRNA interaction	Wang et al., 2020
MD-MLI	miRNA–lncRNA interaction	Song et al., 2020
RPITER	ncRNA–protein interaction	Peng et al., 2019
BGFE	ncRNA–protein interaction	Zhan et al., 2019
DM-RPIs	ncRNA–protein interaction	Cheng et al., 2019
PLRPI	lncRNA–protein interaction	Zhou et al., 2019
CFRP	ncRNA–protein interaction	Dai et al., 2019
McBel-Plnc	lncRNA–protein interaction	Navamajiti et al., 2019
LPI-BLS	lncRNA–protein interaction	Fan and Zhang, 2019
LightGBM	ncRNA–protein interaction	Zhan et al., 2018
IPMiner	ncRNA–protein interaction	Pan et al., 2016
FlaiMapper	small ncRNA identification	Hoogstrate et al., 2015

a *Details of the websites and softwares are listed in Supplementary Table S1*.

### ncRNA Databases

The ncRNA databases collected ncRNA-related information, such as sequences, interactions between ncRNAs and target genes, as well as expression profiles. In addition, some tools contain information that has been supported by experimental evidences. For example, miRbase (Kozomara et al., 2019) integrates published mature sequences of miRNAs and their relevant hairpin precursors of miRNA. miRTarBase (Huang et al., 2020) and NPInter (Teng et al., 2020) not only focus on the interaction information between ncRNAs and other biomolecules, but also provide the relevant experimental evidences. To date, miRNAs, lncRNAs, and circular RNAs (circRNAs) in ncRNAs were collected by different online databases (Chu et al., 2017; Jin et al., 2021). Details of these tools including the application system, input format, and included species are listed in Supplementary Table S1. The development of high-throughput sequencing technologies and the increasing amount of published genomic data have given us the opportunity to collect diverse information from different species, which facilities the analysis of ncRNA evolutional and the construction of conserved models for predicting and exploring novel ncRNAs. However, the application of these databases in cross-species research still faces enormous challenges. On the one hand, there is a lack of identifying species-conserved or species-specific ncRNAs; and on the other hand, it is difficult to determine functional ncRNAs in a predictive manner.

### ncRNA Prediction Tools

Prediction of ncRNA, including recognition of ncRNA and prediction of ncRNA function. At present, there are many prediction tools for ncRNA recognition, but relatively few tools for their function prediction. We classify the existing prediction tools according to their categories (Table 1, Supplementary Table S1). How to accurately predict novel ncRNAs and their target genes has always been the focus of researchers. The current development of prediction tools mainly focuses on three aspects: predicting the sequences of miRNAs and their precursors (Wu et al., 2012; Lei and Sun, 2014; Fei et al., 2021), predicting the binding sites of ncRNAs to targets (Bonnet et al., 2010; Brousse et al., 2014), predicting or visualizing the secondary or three-dimensional structure of ncRNAs (Steffen et al., 2006; Byun and Han, 2009; Biesiada et al., 2016). Sequence alignment is the basis for this prediction, and the divergence of ncRNA sequences is an important factor affecting the accuracy of prediction. In general, ancient ncRNAs (especially those related to plant development) remain highly conserved across species (Willmann and Poethig, 2007). However, recently evolved ncRNAs appear to be highly species-specific (Cuperus et al., 2011). Furthermore, there appears to be variability among different classes of ncRNAs in conservation among different species (Wu et al., 2020). Therefore, how to improve the accuracy of ncRNA prediction is an important problem to be solved. With the accumulation of ncRNA data, building conserved models for each ncRNA family may be a solution for this question.

### Application of Deep Learning in ncRNA Study

Deep learning developed in recent years has shown a powerful potential capability in addressing bioinformatics problems in ncRNA study. For example, RPITER model can be used to predict interactions between ncRNAs and proteins based on sequence and structure information (Peng et al., 2019). Compared with traditional approaches, more information can be introduced into the computational process of deep learning to ensure the accuracy of the predicted results (Zhang S. W. et al., 2020). So far, at least 41 deep learning models have been built to predict ncRNA classification (Amin et al., 2019), ncRNA-protein interaction (Peng et al., 2019), ncRNA interaction, as well as ncRNA identification and functions (Khan et al., 2020; Zhang P. et al., 2020) (Table 1, Supplementary Table S1).

**Figure 1:**
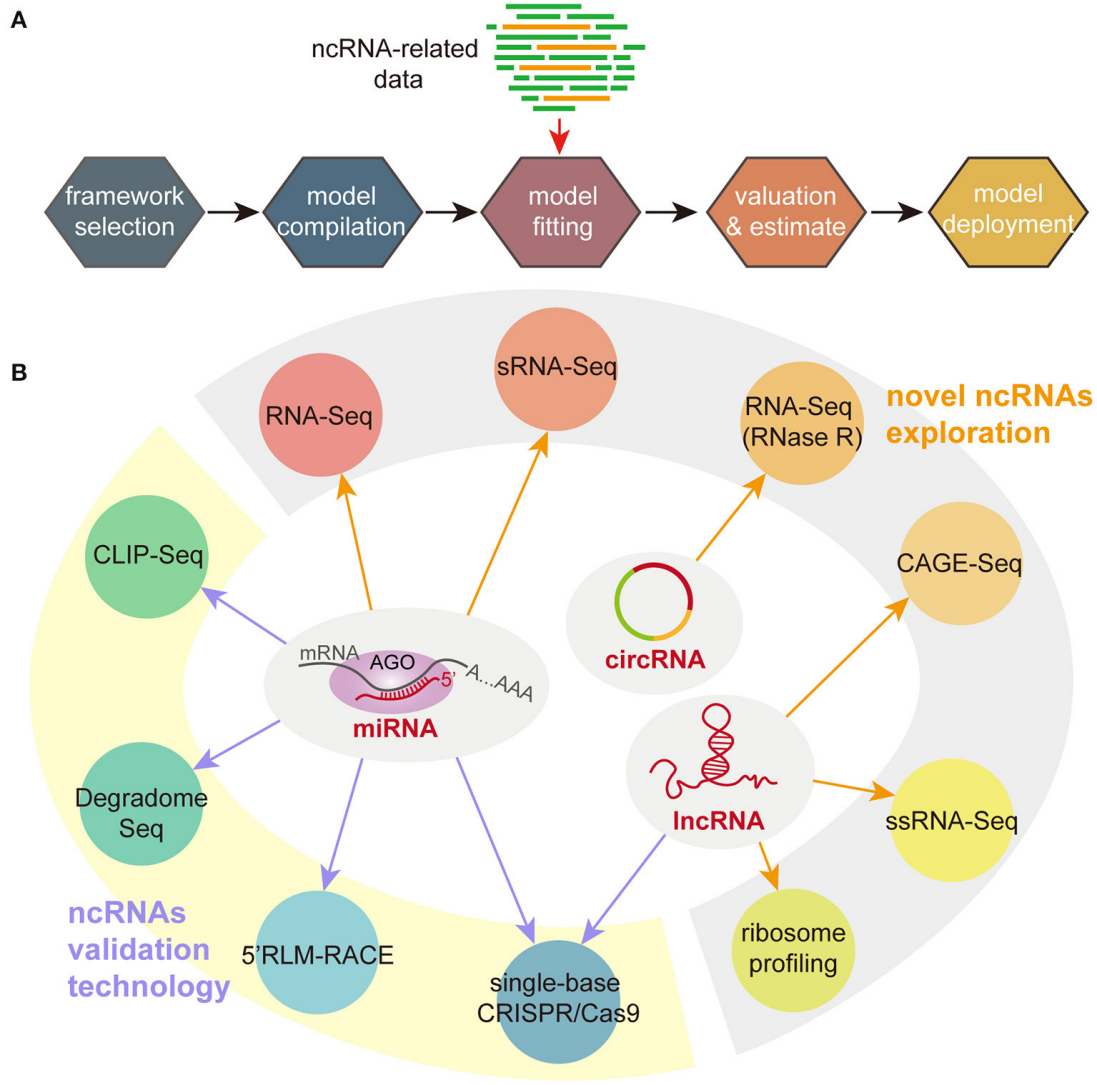
Deep learning processes **(A)** and experimental techniques **(B)** for studying ncRNAs. sRNA-Seq, small RNA sequencing; CLIP-Seq, crosslinking and immunoprecipitation with sequencing; 5′RLM-RACE, 5′ RNA ligase mediated amplification of cDNA ends; ssRNA-Seq, strand-specific RNA sequencing; CAGE-Seq, cap analysis gene expression sequencing.

The basic processes of applying deep learning are showed in Figure 1A. After choosing an appropriate framework, researchers need to input data to generate relevant models. The resulting model can be used for the next prediction step. Compared with other prediction methods, the advantage of deep learning can effectively reduce the prediction bias caused by imperfect design parameters. Its limitation is that the accuracy of predictions heavily depends on the accuracy of the models, which usually require large enough data to build and train. Therefore, the dataset used to build deep learning models seriously affect the accuracy of prediction and analysis. How to obtain a large amount of ncRNA data from different species for building more accurate models is a serious problem that needs to be solved. In addition, most of them are distributed in Linux system, and the proficient computational skills of users are an important precondition to utilize these models. Developing softwares with a user-friendly interface (Xu et al., 2021) is crucial for the application of these models. However, the cross-operating system adaptability of these models and the differences in fit to different data types are the main obstacles facing the use of new models to build user-friendly softwares.

## Experimental Technologies

Although the results of bioinformatics predictions are becoming more and more accurate with the accumulation of ncRNA knowledge, experimental technologies are still needed to further validate the prediction results. According to the characteristics of ncRNAs, at least three aspects need to be verified: firstly, a functional ncRNA should have transcriptional activity (Bazzini et al., 2009); secondly, there should be an expression correlation between ncRNA and target genes (Bai et al., 2018); thirdly, if the ncRNA functions by degrading its target genes, the cleavage site of the ncRNA on the target genes should be verified (Gao et al., 2018). Moreover, experimental strategies should be varied for different types of ncRNAs. Figure 1B summarized the main sequencing and experimental techniques in ncRNA studies. In detail, the combination of sRNA-Seq and RNA-Seq is commonly used for global identification of novel small ncRNAs (Huang et al., 2019). After removed low-quality reads, high-quality reads are subsequently annotated by several databases (such as miRbase and Rfam) (Deforges et al., 2019; Huang et al., 2019). The length of small RNAs is far shorter than protein-coding genes and lncRNAs. Therefore, the extraction of small RNAs unlike other RNA, can be performed using special RNA-extraction kits (Gao et al., 2019) or TRIzol reagent (Tan et al., 2018). For miRNAs, it is necessary to identify the binding sites between miRNAs and their target genes (Shen W. et al., 2021). Degradome sequencing and crosslinking and immunoprecipitation with sequencing (CLIP-Seq) can be used to analyze binding sites between miRNAs and the target genes (Han et al., 2016; Chipman and Pasquinelli, 2019). Furthermore, 5′-RNA ligase mediated amplification of cDNA ends (5′RLM-RACE) assays are directly used for verifying the predicted binding sites (Cui et al., 2020). The detection of novel lncRNAs can be completed by multiple RNA-Seq strategies, such as isoform-sequencing, strand-specific RNA-Seq (ssRNA-Seq), cap analysis gene expression with polyA-Seq technologies (CAGE-Seq) (Zheng et al., 2021). Although lncRNAs are considered as a part of ncRNAs, some of them still remain a weak ability of translating small peptides. Therefore, ribosome profiling become has become one of the strategies to detect lncRNAs (Wu et al., 2019). Meanwhile, the recently developed high-precision single-base CRISPR/Cas9 technology can effectively create ncRNA-related mutants to explore the relationships between ncRNAs and target genes (Jacobs et al., 2015). Currently, in addition to the research on the function of small RNAs, more and more attention is focused on circRNAs in recent years. However, most studies focus more on the discovery of novel circRNAs by sequencing technologies. One of challenges of circRNA sequencing is to improve the accuracy of detection and quantification of circRNAs due to their lack of poly (A) tails and insufficient expression levels (Wang et al., 2019; Zhao et al., 2019). Treatment of total RNA with ribonuclease R or increasing sequencing depth may resolve these issues (Chen et al., 2017). Meanwhile, it is also necessary to develop functional research techniques to further clarify the biological functions of circRNAs.

## Conclusion

In conclusion, although many tools and technologies have been developed to study ncRNAs in plants, there are still opportunities and challenges in this field. In bioinformatics, since there are significant differences in ncRNAs between species, it is beneficial for our research on ncRNAs to collect as much data as possible based on different species. Meanwhile, ncRNAs in a same family exhibit high conservation, it is possible for us to build models to discover novel ncRNAs. Moreover, most prediction tools and deep-learning models are developed based on Linux system, and the development of user-friendly Windows versions will help more researchers to analyze different kinds of ncRNA. As ncRNAs play a regulatory role in plants, how to manipulate ncRNAs through genetic engineering to regulate specific biological processes remains to be resolved.

## Author Contributions

JZ conceived the study. DX collected and synthesized the data and draft the manuscript. JZ, WY, CF, BL, and M-ZL revised the manuscript. All authors contributed to the article and approved the final version.

## Funding

This work was supported by the Key Scientific and Technological Grant of Zhejiang for Breeding New Agricultural Varieties (2021C02070-1), the National Key Research and Development Program of China (2021YFD2200205 and 2021YFD2200700), the National Science Foundation of China (32171814), the Natural Science Foundation of Zhejiang Province for Distinguished Young Scholars (LR22C160001), and the Zhejiang A&F University Research and Development Fund Talent Startup Project (2021LFR013).

## Conflict of Interest

The authors declare that the research was conducted in the absence of any commercial or financial relationships that could be construed as a potential conflict of interest.

## Publisher's Note

All claims expressed in this article are solely those of the authors and do not necessarily represent those of their affiliated organizations, or those of the publisher, the editors and the reviewers. Any product that may be evaluated in this article, or claim that may be made by its manufacturer, is not guaranteed or endorsed by the publisher.

## References

[B1] Amin N. McGrath A. Chen Y.-P. P. (2019). Evaluation of deep learning in non-coding RNA classification. Nat. Machine Intellig. 1, 246–256. 10.1038/s42256-019-0051-2

[B2] Bai Q. Wang X. Chen X. Shi G. Liu Z. Guo C.. (2018). Wheat miRNA TaemiR408 acts as an essential mediator in plant tolerance to pi deprivation and salt stress *via* modulating stress-associated physiological processes. Front. Plant Sci. 9, 499. 10.3389/fpls.2018.00499 29720988 PMC5916090

[B3] Baum J. A. Bogaert T. Clinton W. Heck G. R. Feldmann P. Ilagan O.. (2007). Control of coleopteran insect pests through RNA interference. Nat. Biotechnol. 25, 1322–1326. 10.1038/nbt1359 17982443

[B4] Bazzini A. A. Almasia N. I. Manacorda C. A. Mongelli V. C. Conti G. Maroniche G. A.. (2009). Virus infection elevates transcriptional activity of miR164a promoter in plants. BMC Plant Biol. 9, 152. 10.1186/1471-2229-9-152 20042107 PMC2809068

[B5] Betti F. Ladera-Carmona M. J. Weits D. A. Ferri G. Iacopino S. Novi G.. (2021). Exogenous miRNAs induce post-transcriptional gene silencing in plants. Nature Plants 7, 1379–1388. 10.1038/s41477-021-01005-w 34650259 PMC8516643

[B6] Biesiada M. Purzycka K. J. Szachniuk M. Blazewicz J. Adamiak R. W. (2016). “Automated RNA 3D structure prediction with RNAComposer,” in RNA Structure Determination (New York, NY: Humana Press), 199–215. 10.1007/978-1-4939-6433-8_13 27665601

[B7] Bonnet E. He Y. Billiau K. Van de Peer Y. (2010). TAPIR, a web server for the prediction of plant microRNA targets, including target mimics. Bioinformatics 26, 1566–1568. 10.1093/bioinformatics/btq233 20430753

[B8] Brousse C. Liu Q. Beauclair L. Deremetz A. Axtell M. J. Bouché N. (2014). A non-canonical plant microRNA target site. Nucleic Acids Res. 42, 5270–5279. 10.1093/nar/gku157 24561804 PMC4005643

[B9] Byun Y. Han K. (2009). PseudoViewer3: generating planar drawings of large-scale RNA structures with pseudoknots. Bioinformatics 25, 1435–1437. 10.1093/bioinformatics/btp252 19369500

[B10] Chaabane M. Williams R. M. Stephens A. T. Park J. W. (2020). circDeep: deep learning approach for circular RNA classification from other long non-coding RNA. Bioinformatics 36, 73–80. 10.1093/bioinformatics/btz537 31268128 PMC6956777

[B11] Chantsalnyam T. Lim D. Y. Tayara H. Chong K. T. (2020). ncRDeep: non-coding RNA classification with convolutional neural network. Comput. Biol. Chem. 88, 107364. 10.1016/j.compbiolchem.2020.107364 32890916

[B12] Chantsalnyam T. Siraj A. Tayara H. Chong K. T. (2021). ncRDense: a novel computational approach for classification of non-coding RNA family by deep learning. Genomics 113, 3030–3038. 10.1016/j.ygeno.2021.07.004 34242708

[B13] Charrier A. Vergne E. Joffrion C. Richer A. Dousset N. Chevreau E. (2019). An artificial miRNA as a new tool to silence and explore gene functions in apple. Transgenic Res. 28, 611–626. 10.1007/s11248-019-00170-1 31538273

[B14] Chen G. Cui J. Wang L. Zhu Y. Lu Z. Jin B. (2017). Genome-wide identification of circular RNAs in Arabidopsis thaliana. Front. Plant Sci. 8, 1678. 10.3389/fpls.2017.01678 29021802 PMC5623955

[B15] Chen Q. J. Deng B. H. Gao J. Zhao Z. Y. Chen Z. L. Song S. R.. (2020). A miRNA-encoded small peptide, vvi-miPEP171d1, regulates adventitious root formation. Plant Physiol. 183, 656–670. 10.1104/pp.20.00197 32241877 PMC7271809

[B16] Cheng S. Zhang L. Tan J. Gong W. Li C. Zhang X. (2019). DM-RPIs: predicting ncRNA-protein interactions using stacked ensembling strategy. Comput. Biol. Chem. 83, 107088. 10.1016/j.compbiolchem.2019.107088 31330489

[B17] Chipman L. B. Pasquinelli A. E. (2019). miRNA targeting: growing beyond the seed. Trends Genet. 35, 215–222. 10.1016/j.tig.2018.12.005 30638669 PMC7083087

[B18] Chu Q. Zhang X. Zhu X. Liu C. Mao L. Ye C.. (2017). PlantcircBase: a database for plant circular RNAs. Mol. Plant 10, 1126–1128. 10.1016/j.molp.2017.03.003 28315753

[B19] Cui C. Wang J. J. Zhao J. H. Fang Y. Y. He X. F. Guo H. S.. (2020). A brassica miRNA regulates plant growth and immunity through distinct modes of action. Mol. Plant 13, 231–245. 10.1016/j.molp.2019.11.010 31794845

[B20] Cuperus J. T. Fahlgren N. Carrington J. C. (2011). Evolution and functional diversification of MIRNA genes. Plant Cell 23, 431–442. 10.1105/tpc.110.082784 21317375 PMC3077775

[B21] Dai Q. Guo M. Duan X. Teng Z. Fu Y. (2019). Construction of complex features for computational predicting ncRNA-protein interaction. Front. Genet. 10, 18. 10.3389/fgene.2019.00018 30774646 PMC6367266

[B22] Dai Q. Wang Z. Song J. Duan X. Guo M. Tian Z. (2020). “A stacked ensemble learning framework with heterogeneous feature combinations for predicting ncRNA-protein interaction,” in 2020 IEEE International Conference on Bioinformatics and Biomedicine (Seoul).

[B23] Deforges J. Reis R. S. Jacquet P. Sheppard S. Gadekar V. P. Hart-Smith G.. (2019). Control of cognate sense mRNA translation by cis-natural antisense RNAs. Plant Physiol. 180, 305–322. 10.1104/pp.19.00043 30760640 PMC6501089

[B24] Fan S. Zhang D. Gao C. Wan S. Lei C. Wang J.. (2018). Mediation of flower induction by gibberellin and its inhibitor paclobutrazol: mRNA and miRNA integration comprises complex regulatory cross-talk in apple. Plant Cell Physiol. 59, 2288–2307. 10.1093/pcp/pcy154 30137602

[B25] Fan X. N. Zhang S. W. (2019). LPI-BLS: Predicting lncRNA?protein interactions with a broad learning system-based stacked ensemble classifier. Neurocomputing 370, 88–93. 10.1016/j.neucom.2019.08.084

[B26] Fasold M. Langenberger D. Binder H. Stadler P. F. Hoffmann S. (2011). DARIO: a ncRNA detection and analysis tool for next-generation sequencing experiments. Nucleic Acids Res. 39, W112–W117. 10.1093/nar/gkr357 21622957 PMC3125765

[B27] Fei Y. Feng J. Wang R. Zhang B. Zhang H. Huang J. (2021). PhasiRNAnalyzer: an integrated analyser for plant phased siRNAs. RNA Biology 18, 1622–1629. 10.1080/15476286.2021.1879543 33541212 PMC8594884

[B28] Fiannaca A. La Rosa M. La Paglia L. Rizzo R. Urso A. (2017). nRC: non-coding RNA Classifier based on structural features. BioData Min. 10, 27. 10.1186/s13040-017-0148-2 28785313 PMC5540506

[B29] Gao J. Chen H. Yang H. He Y. Tian Z. Li J. (2018). A brassinosteroid responsive miRNA-target module regulates gibberellin biosynthesis and plant development. New Phytol. 220, 488–501. 10.1111/nph.15331 30009574

[B30] Gao Z. Li J. Luo M. Li H. Chen Q. Wang L.. (2019). Characterization and cloning of grape circular RNAs identified the cold resistance-related *Vv-circATS1*. Plant Physiol. 180, 966–985. 10.1104/pp.18.01331 30962290 PMC6548266

[B31] Han X. Yin H. Song X. Zhang Y. Liu M. Sang J.. (2016). Integration of small RNAs, degradome and transcriptome sequencing in hyperaccumulator *Sedum alfredii* uncovers a complex regulatory network and provides insights into cadmium phytoremediation. Plant Biotechnol. J. 14, 1470–1483. 10.1111/pbi.12512 26801211 PMC5066797

[B32] Hoogstrate Y. Jenster G. Martens-Uzunova E. S. (2015). FlaiMapper: computational annotation of small ncRNA-derived fragments using RNA-seq high-throughput data. Bioinformatics 31, 665–673. 10.1093/bioinformatics/btu696 25338717

[B33] Huang H. Y. Lin Y. C. D. Li J. Huang K. Y. Shrestha S.. (2020). miRTarBase 2020: updates to the experimentally validated microRNA-target interaction database. Nucleic Acids Res. 48, D148–D154, 10.1093/nar/gkz896 31647101 PMC7145596

[B34] Huang J. Wang C. Li X. Fang X. Huang N. Wang Y.. (2019). Conservation and divergence in the meiocyte sRNAomes of *Arabidopsis*, soybean, and cucumber. Plant Physiol. 182, 301–317. 10.1104/pp.19.00807 31719152 PMC6945826

[B35] Huang L. Jiao S. Yang S. Zhang S. Zhu X. Guo R.. (2021). LGFC-CNN: prediction of lncRNA-protein interactions by using multiple types of features through deep learning. Genes 12, 1689. 10.3390/genes12111689 34828296 PMC8621699

[B36] Jacobs T. B. LaFayette P. R. Schmitz R. J. Parrott W. A. (2015). Targeted genome modifications in soybean with CRISPR/Cas9. BMC Biotechnology 15, 16. 10.1186/s12896-015-0131-2 25879861 PMC4365529

[B37] Jin J. Lu P. Xu Y. Li Z. Yu S. Liu J.. (2021). PLncDB V2. 0: a comprehensive encyclopedia of plant long noncoding RNAs. Nucleic Acids Res. 49, D1489–D1495. 10.1093/nar/gkaa910 33079992 PMC7778960

[B38] Kang Q. Meng J. Cui J. Luan Y. Chen M. (2020). PmliPred: a method based on hybrid model and fuzzy decision for plant miRNA–lncRNA interaction prediction. Bioinformatics 36, 2986–2992. 10.1093/bioinformatics/btaa074 32087005

[B39] Kang Q. Meng J. Shi W. Luan Y. (2021). Ensemble deep learning based on multi-level information enhancement and greedy fuzzy decision for plant miRNA–lncRNA interaction prediction. Interdiscip. Sci. Comput. Life Sci. 13, 603–614. 10.1007/s12539-021-00434-7 33900552

[B40] Khan S. Khan M. Iqbal N. Hussain T. Khan S. A. Chou K. C. (2020). A two-level computation model based on deep learning algorithm for identification of piRNA and their functions *via* Chou's 5-steps rule. Int. J. Peptide Res.and Therap. 26, 795–809. 10.1007/s10989-019-09887-3

[B41] Kozomara A. Birgaoanu M. Griffiths-Jones S. (2019). miRBase: from microRNA sequences to function. Nucleic Acids Res. 47, D155–D162. 10.1093/nar/gky1141 30423142 PMC6323917

[B42] Lai X. L. Jérémie B. Stuart W. Martin C. Chloe Z. Simon J. C. (2018). CircRNAs in plants. Circular RNAs. Adv. Exp. Med. Biol. (Springer), 1087, 329–343. 10.1007/978-981-13-1426-1_26 30259378

[B43] Lei J. Sun Y. (2014). miR-PREFeR: an accurate, fast and easy-to-use plant miRNA prediction tool using small RNA-Seq data. Bioinformatics 30, 2837–2839. 10.1093/bioinformatics/btu380 24930140

[B44] Li Y. Sun H. Feng S. Zhang Q. Han S. Du W. (2021). Capsule-LPI: a lncRNA–protein interaction predicting tool based on a capsule network. BMC Bioinformatics 22, 246. 10.1186/s12859-021-04171-y 33985444 PMC8120853

[B45] Liu S. Zhao X. Zhang G. Li W. Liu F. Liu S.. (2019). PredLnc-GFStack: a global sequence feature based on a stacked ensemble learning method for predicting lncRNAs from transcripts. Genes 10, 672. 10.3390/genes10090672 31484412 PMC6770532

[B46] Liu X. Hao L. Li D. Zhu L. Hu S. (2015). Long non-coding RNAs and their biological roles in plants. Genom. Proteom. Bioinform. 13, 137–147. 10.1016/j.gpb.2015.02.003 25936895 PMC4563214

[B47] Miller W. A. Shen R. Staplin W. Kanodia P. (2016). Noncoding RNAs of plant viruses and viroids: sponges of host translation and RNA interference machinery. Mol. Plant Microbe Interact. 29, 156–164. 10.1094/MPMI-10-15-0226-FI 26900786 PMC5410770

[B48] Navamajiti N. Saethang T. Wichadakul D. (2019). “McBel-Plnc: a deep learning model for multiclass multilabel classification of protein-lncRNA interactions,” in Proceedings of the 2019 6th International Conference on Biomedical and Bioinformatics Engineering. Association for Computing Machinery, Shanghai, China, 21–28.

[B49] Pan X. Fan Y. X. Yan J. Shen H. B. (2016). IPMiner: hidden ncRNA-protein interaction sequential pattern mining with stacked autoencoder for accurate computational prediction. BMC Genomics 17, 582. 10.1186/s12864-016-2931-8 27506469 PMC4979166

[B50] Peng C. Han S. Zhang H. Li Y. (2019). RPITER: a hierarchical deep learning framework for ncRNA–protein interaction prediction. Int. J. Mol. Sci. 20, 1070. 10.3390/ijms20051070 30832218 PMC6429152

[B51] Ramakrishnaiah Y. Kuhlmann L. Tyagi S. (2021). linc2function: a deep learning model to identify and assign function to long noncoding RNA. bioRxiv [Preprint]. 10.1101/2021.01.29.428785

[B52] Shaw D. Chen H. Xie M. Jiang T. (2021). DeepLPI: a multimodal deep learning method for predicting the interactions between lncRNAs and protein isoforms. BMC Bioinform. 22, 24. 10.1186/s12859-020-03914-7 33461501 PMC7814738

[B53] Shen W. Cao S. Liu J. Zhang W. Chen J. Li J. F. (2021). Overexpression of an Osa-miR162a derivative in rice confers cross-kingdom RNA interference-mediated brown planthopper resistance without perturbing host development. Int. J. Mol. Sci. 22, 12652. 10.3390/ijms222312652 34884461 PMC8657652

[B54] Shen Z. A. Luo T. Zhou Y. K. Yu H. Du P. F. (2021). NPI-GNN: predicting ncRNA–protein interactions with deep graph neural networks. Brief. Bioinform. 22, bbab051. 10.1093/bib/bbab051 33822882

[B55] Song J. Tian S. Yu L. Yang Q. Xing Y. Zhang C.. (2020). MD-MLI: prediction of miRNA-lncRNA interaction by using multiple features and hierarchical deep learning. IEEE/ACM Trans. Comput. Biol. Bioinform. 10.1109/TCBB.2020.3034922. [Epub ahead of print]. 33125334

[B56] Steffen P. Voß B. Rehmsmeier M. Reeder J. Giegerich R. (2006). RNAshapes: an integrated RNA analysis package based on abstract shapes. Bioinformatics 22, 500–503. 10.1093/bioinformatics/btk010 16357029

[B57] Tan F. Lu Y. Jiang W. Wu T. Zhang R. Zhao Y.. (2018). DDM1 represses noncoding RNA expression and RNA-directed DNA methylation in heterochromatin. Plant Physiol. 177, 1187–1197. 10.1104/pp.18.00352 29794169 PMC6052999

[B58] Tang Q. Nie F. Kang J. Chen W. (2020). ncPro-ML: an integrated computational tool for identifying non-coding RNA promoters in multiple species. Comput. Struct. Biotechnol. J. 18, 2445–2452. 10.1016/j.csbj.2020.09.001 33005306 PMC7509369

[B59] Teng X. Chen X. Xue H. Tang Y. Zhang P. Kang Q.. (2020). NPInter v4.0: an integrated database of ncRNA interactions. Nucleic Acids Res. 48, D160–D165. 10.1093/nar/gkz969 31670377 PMC7145607

[B60] Wang J. Zhao Y. Gong W. Liu Y. Wang M. Huang X.. (2021a). EDLMFC: an ensemble deep learning framework with multi-scale features combination for ncRNA–protein interaction prediction. BMC Bioinformatics 22, 133. 10.1186/s12859-021-04069-9 33740884 PMC7980572

[B61] Wang L. Zhong X. Wang S. Liu Y. (2021b). ncDLRES: a novel method for non-coding RNAs family prediction based on dynamic LSTM and ResNet. BMC Bioinformatics 22, 447. 10.1186/s12859-021-04365-4 34544356 PMC8451086

[B62] Wang W. Guan X. Khan M. T. Xiong Y. Wei D. Q. (2020). LMI-DForest: a deep forest model towards the prediction of lncRNA-miRNA interactions. Comput. Biol. Chem. 89, 107406. 10.1016/j.compbiolchem.2020.107406 33120126

[B63] Wang Y. Wang X. Deng W. Fan X. Liu T. T. He G.. (2014). Genomic features and regulatory roles of intermediate-sized non-coding RNAs in *Arabidopsis*. Mol. Plant 7, 514–527. 10.1093/mp/sst177 24398630

[B64] Wang Y. Xiong Z. Li Q. Sun Y. Y. Jin J. Chen H.. (2019). Circular RNA profiling of the rice photo-thermosensitive genic male sterile line Wuxiang S reveals circRNA involved in the fertility transition. BMC Plant Biol. 19, 340. 10.1186/s12870-019-1944-2 31382873 PMC6683460

[B65] Wekesa J. S. Meng J. Luan Y. (2020b). A deep learning model for plant lncRNA-protein interaction prediction with graph attention. Mol. Genet. Genom. 295, 1091–1102. 10.1007/s00438-020-01682-w 32409904

[B66] Wekesa J. S. Meng J. Luan Y. (2020c). Multi-feature fusion for deep learning to predict plant lncRNA-protein interaction. Genomics 112, 2928–2936. 10.1016/j.ygeno.2020.05.005 32437848

[B67] Wekesa J. S. Luan Y. Meng J. (2020a). “LPI-DL: a recurrent deep learning model for plant lncRNA-protein interaction and function prediction with feature optimization,” in 2020 IEEE International Conference on Bioinformatics and Biomedicine (Seoul).

[B68] Willmann M. R. Poethig R. S. (2007). Conservation and evolution of miRNA regulatory programs in plant development. Curr. Opin. Plant Biol. 10, 503–511. 10.1016/j.pbi.2007.07.004 17709279 PMC2080797

[B69] Wu H. J. Ma Y. K. Chen T. Wang M. Wang X. J. (2012). PsRobot: a web-based plant small RNA meta-analysis toolbox. Nucleic Acids Res. 40, W22–W28. 10.1093/nar/gks554 22693224 PMC3394341

[B70] Wu H. Y. L. Song G. Walley J. W. Hsu P. Y. (2019). The tomato translational landscape revealed by transcriptome assembly and ribosome profiling. Plant Physiol. 181, 367–380. 10.1104/pp.19.00541 31248964 PMC6716236

[B71] Wu L. Liu S. Qi H. Cai H. Xu M. (2020). Research progress on plant long non-coding RNA. Plants 9, 408. 10.3390/plants9040408 32218186 PMC7237992

[B72] Xu D. Lu Z. Jin K. Qiu W. Qiao G. Han X.. (2021). SPDE: a multi-functional software for sequence processing and data extraction. Bioinformatics 37, 3686–3687. 10.1093/bioinformatics/btab235 33848326

[B73] Yang C. Yang L. Zhou M. Xie H. Zhang C. Wang M. D.. (2018). LncADeep: an ab initio lncRNA identification and functional annotation tool based on deep learning. Bioinformatics 34, 3825–3834. 10.1093/bioinformatics/bty428 29850816

[B74] Yang S. Wang Y. Lin Y. Shao D. He K. Huang L. (2020). LncMirNet: predicting lncRNA–miRNA interaction based on deep learning of ribonucleic acid sequences. Molecules 25, 4372. 10.3390/molecules25194372 32977679 PMC7583909

[B75] Yi H. C. You Z. H. Wang M. N. Guo Z. H. Wang Y. B. Zhou J. R. (2020). RPI-SE: a stacking ensemble learning framework for ncRNA-protein interactions prediction using sequence information. BMC Bioinformatics 21, 60. 10.1186/s12859-020-3406-0 32070279 PMC7029608

[B76] Yogindran S. Rajam M. V. (2021). Host-derived artificial miRNA-mediated silencing of ecdysone receptor gene provides enhanced resistance to *Helicoverpa armigera* in tomato. Genomics 113, 736–747. 10.1016/j.ygeno.2020.10.004 33058987

[B77] Yu H. Shen Z. A. Du P. F. (2021). NPI-RGCNAE: fast predicting ncRNA-protein interactions using the relational graph convolutional network auto-encoder. IEEE J. Biomed. Health Inform. 10.1109/JBHI.2021.3122527. [Epub ahead of print]. 34699377

[B78] Zhan Z. H. Jia L. N. Zhou Y. Li L. P. Yi H. C. (2019). BGFE: a deep learning model for ncRNA-protein interaction predictions based on improved sequence Information. Int. J. Mol. Sci. 20, 978. 10.3390/ijms200409783 30813451 PMC6412311

[B79] Zhan Z. H. You Z. H. Li L. P. Zhou Y. Yi H. C. (2018). Accurate prediction of ncRNA-protein interactions from the integration of sequence and evolutionary information. Front. Genet. 9, e00458. 10.3389/fgene.2018.00458 30349558 PMC6186793

[B80] Zhang P. Meng J. Luan Y. Liu C. (2020). Plant miRNA–lncRNA interaction prediction with the ensemble of CNN and IndRNN. Interdiscipl. Sci. Comput. Life Sci. 12, 82–89. 10.1007/s12539-019-00351-w 31811618

[B81] Zhang S. W. Zhang X. X. Fan X. N. Li W. N. (2020). LPI-CNNCP: prediction of lncRNA-protein interactions by using convolutional neural network with the copy-padding trick. Anal. Biochem. 601, 113767. 10.1016/j.ab.2020.113767 32454029

[B82] Zhang Y. Jia C. Kwoh C. K. (2021). Predicting the interaction biomolecule types for lncRNA: an ensemble deep learning approach. Brief. Bioinform. 22, bbaa228. 10.1093/bib/bbaa228 33003205

[B83] Zhao W. Chu S. S. Jiao Y. Q. (2019). Present scenario of circular RNAs (circRNAs) in plants. Front. Plant Sci. 10, 379. 10.3389/fpls.2019.00379 31001302 PMC6454147

[B84] Zheng X. Chen Y. Zhou Y. Shi K. Hu X. Li D.. (2021). Full-length annotation with multistrategy RNA-seq uncovers transcriptional regulation of lncRNAs in cotton. Plant Physiol. 185, 179–195. 10.1093/plphys/kiaa003 33631798 PMC8133545

[B85] Zhou H. Wekesa J. S. Luan Y. Meng J. (2021). PRPI-SC: an ensemble deep learning model for predicting plant lncRNA-protein interactions. BMC Bioinformatics 22, 415. 10.1186/s12859-021-04328-9 34429059 PMC8385908

[B86] Zhou H. Luan Y. Wekesa J. S. Meng J. (2019). “Prediction of plant lncRNA-protein interactions using sequence information based on deep learning,” in International Conference on Intelligent Computing, Springer (Cham), 358–368.

